# On-Chip Inverted Emulsion Method for Fast Giant Vesicle Production, Handling, and Analysis

**DOI:** 10.3390/mi11030285

**Published:** 2020-03-10

**Authors:** Naresh Yandrapalli, Tina Seemann, Tom Robinson

**Affiliations:** Department of Theory & Bio-Systems, Max Planck Institute of Colloids and Interfaces, Science Park Golm, 14424 Potsdam, Germany

**Keywords:** lab-on-chip, inverted emulsion method, microfluidics, giant vesicles, giant unilamellar vesicles (GUVs), bottom-up synthetic biology

## Abstract

Liposomes and giant unilamellar vesicles (GUVs) in particular are excellent compartments for constructing artificial cells. Traditionally, their use requires bench-top vesicle growth, followed by experimentation under a microscope. Such steps are time-consuming and can lead to loss of vesicles when they are transferred to an observation chamber. To overcome these issues, we present an integrated microfluidic chip which combines GUV formation, trapping, and multiple separate experiments in the same device. First, we optimized the buffer conditions to maximize both the yield and the subsequent trapping of the vesicles in micro-posts. Captured GUVs were monodisperse with specific size of 18 ± 4 µm in diameter. Next, we introduce a two-layer design with integrated valves which allows fast solution exchange in less than 20 s and on separate sub-populations of the trapped vesicles. We demonstrate that multiple experiments can be performed in a single chip with both membrane transport and permeabilization assays. In conclusion, we have developed a versatile all-in-one microfluidic chip with capabilities to produce and perform multiple experiments on a single batch of vesicles using low sample volumes. We expect this device will be highly advantageous for bottom-up synthetic biology where rapid encapsulation and visualization is required for enzymatic reactions.

## 1. Introduction

Reproducing complex cellular systems in a controlled manner is at the forefront of bottom-up synthetic biology [1,2]. Techniques such as gentle hydration [3,4] and electroformation [5,6] are very well established for producing lipid-based vesicles that are commonly used as artificial cells [7,8]. Lesser used techniques such as the inverted emulsion method [9,10] or microfluidics [11,12] are being actively developed due to their abilities to encapsulate large biomolecules inside lipid vesicles while still maintaining physiological buffers—both of which are important requirements in realizing the dream of a functioning artificial cell [13,14,15,16]. Despite their clear advantages, these methods have limitations which restricts the experimental possibilities.

A typical experiment involving giant unilamellar vesicles (GUVs) includes preparation and transfer to a pre-coated observation chamber, followed by experimentation/visualization under a microscope. This is a lengthy process which is not always suitable for time-sensitive experiments, i.e., encapsulation of enzymatic reactions. In addition, during the harvesting and transfer process, care must be taken to avoid bursting or leakage as this can result in the loss of a sub-population of GUVs and lead to misrepresentative results. In the case of vesicles prepared via the inverted emulsion method, contamination by the oil phase during the harvesting step presents an additional challenge. While methods such as careful removal of the top oil layer with successive centrifugation steps [17] or the use of a syringe to pierce the bottom of the Eppendorf [18] can be used, they introduce additional steps and can still lead to loss of vesicles.

Even after successful transfer to an observation chamber, continuous imaging for long time periods while adding or removing external components is challenging. Batch-to-batch variations, which are inherent to GUV preparation techniques, also require repeat experiments to avoid misleading datasets. Many researchers have used microfluidics to overcome some of these issues by trapping and observing vesicles inside various devices [2,19,20,21]. Our previous work and others have shown that it is possible to trap GUVs in microfluidic channels with the aid of micrometer-sized features such as posts or wells [21,22]. Recently, we introduced a high-throughput device which reduces within-batch variations by trapping large numbers of GUVs from the same preparation [23]. Some researchers have taken this one step further by performing vesicle production within microfluidic chips [24,25,26,27] and even trapping them on the same platform for visualization and analysis [25,28,29]. Combining production and experimentation in a single platform is highly advantageous as it reduces the overall time from formation to observation and minimizes loss of sample. However, the double-emulsion template method, which is considered the gold standard for on-chip production, is non-trivial and requires complex setups [30,31,32,33].

In this communication, we present a straightforward microfluidic method to produce GUVs via an on-chip inverted emulsion method. The resulting vesicles are then directly trapped using micro-posts within the same device for observation and analysis. First, we optimize the buffer conditions to maximize the yield of GUVs. Then, we characterize the size of vesicles before and after trapping, as well the occupation efficiency. Furthermore, we show that the addition of more than one inlet prevents the need to remove oil present in the reservoir where the inverted emulsion method is performed. To increase the versatility of the device, sets of vesicles are isolated from each other with the help of microfluidic valves, and multiple experiments are performed by solution exchange on specific sub-populations. This approach not only addresses the aforementioned time factor but also prevents batch-to-batch variations, as multiple conditions can be applied to the same preparation of vesicles. After fluidic exchange is demonstrated using fluorescent dyes, different tests are carried out on the same device, demonstrating its capability to perform multiple experiments. The use of on-chip vesicle production via the inverted emulsion method opens up the possibility to encapsulate large biomolecules such as enzymes, which is essential for the bottom-up creation of artificial cells.

## 2. Materials and Methods

### 2.1. Materials and Equipment

1-Palmitoyl-2-oleoyl-glycero-3-phosphocholine (POPC) was purchased from Avanti Polar Lipids (Alabaster, AL, USA). 1,1′-Dioctadecyl-3,3,3′,3′-tetramethylindocarbocyanine-5,5′-disulfonic acid (DiIC_18_), 4′,6-Diamidino-2-phenylindole dihydrochloride (DAPI), and calcein were purchased from ThermoFisher Scientific (Waltham, MA, USA). Chloroform used for dissolving and storing lipid stocks was purchased from Merck (Darmstadt, Germany) along with other reagents: glucose, sucrose, Triton X-100, and light mineral oil (catalog code: 330779-1L). β-Casein and bovine serum albumin (BSA) used to coat the glass coverslips were purchased from Sigma-Aldrich (Taufkirchen, Germany). Materials for producing microfluidics chips, polydimethylsiloxane (PDMS) and curing agent, were purchased as the SYLGARD^®^ 184 silicone elastomer kit from Dow Corning (Midland, MI, USA). 1*H*,1*H*,2*H*,2*H*-Perfluorodecyltrichlorosilane was purchased from abcr GmbH (Karlsruhe, Germany). Materials for the dye leakage assay, fluorescein and α-hemolysin (α-HL), were purchased from Sigma. Millipore^®^ MilliQ water was used to make all the aqueous solutions. Pressure was applied to the chip using a nitrogen gas line via a custom-built device. Details of the operation can be found elsewhere [34].

### 2.2. Solutions for the On-Chip Inverted Emulsion Method

A lipid–oil solution was made by drying 75 µL of 200 mM lipid mixture POPC:DiIC_18_ (99.5:0.5%) in chloroform under argon flow. Residual chloroform was removed by desiccation for a period of 45 min. Mineral oil was added to the dried lipid film to make the total lipid concentration of 180 µM. The vial containing the mixture was kept under bath sonication for a period of 30 min in degassing mode to fully solubilize the lipids.

Sucrose and glucose sugar solutions, which were used to create a density gradient, were made by dissolving respective amounts in MilliQ water to generate various concentrations: 100, 300, 600, and 900 mM.

### 2.3. Microfabrication

Fabrication of the microfluidic devices was performed using a procedure described previously [21]. Briefly, four-inch silicon wafers (Si-Mat, Kaufering, Germany) with the required design were produced via soft photolithography to a height of 20 μm. Silicon wafers coated with SU8 3025 (Microchem, Newton, MA, USA) were exposed to UV light through a film mask (Micro Lithography Services, Chelmsford, UK) and un-exposed photoresist was removed. Salinization was performed by overnight incubation with 50 μL of 1*H*,1*H*,2*H*,2*H*-perfluorodecyltrichlorosilane in a desiccator. PDMS-based chips were produced by pouring the PDMS/curing agent mixture (10:1) on top of the silanized wafer, and then curing at 90 °C for three hours. Cured PDMS was removed from the master mold, and holes were punched at dedicated inlets and outlets using a 1.5-mm biopsy puncher (Kai Europe, Solingen, Germany). This was followed by bonding to pre-cleaned glass coverslips using an air plasma treatment (Plasma Cleaner PDC- 002-CE, Harrick Plasma, Ithaca, NY, USA) at 0.6 bar for 1 min. Two small reservoirs made from 200 μL pipette tips, with capacities of 150 μL, were glued to the two inlets with PDMS and cured for a period of 2 h at 80 °C. In the case of double-layer chips with valves, the top layer with the circular valve design was produced similar to the procedure mentioned above. Instead of bonding to the coverslip, the chips with top-layer design with 1-mm punched holes were bonded to the bottom fluidic layer prepared by curing 40 µm height spin-coated PDMS on the corresponding master mold. After successful alignment of the two layers, both were removed from the silicon wafer and 1.5 mm inlets and an outlet were punched. Final chips were prepared by bonding to glass coverslips and the creation of fluidic reservoirs as above. Note that, unlike in previous works [21], this new design comprises two inlets to separate vesicle formation from the new solutions, 108 traps, and a modification of the trap dimensions to capture larger GUVs (see results section).

### 2.4. Microscopy

Images of the GUVs were taken using a confocal microscope (SP8, Leica microsystems, Weizlar, Germany). Calcein was imaged with an excitation wavelength of 488 nm, and the emission was collected between 498 nm and 540 nm. For DiIC_18_, the excitation wavelength was set to 551 nm and emission was collected between 562 nm and 628 nm. DAPI was excited with 405 nm, and emission was collected from 415 to 440 nm. For bright-field imaging of the microfluidic chip in color, an epifluorescence microscope was used (AZ100, Nikon, Tokyo, Japan). Image analysis was conducted using Fiji (ImageJ, National Institutes of Health, Bethesda, MD, USA).

## 3. Results and Discussion

### 3.1. Chip Design and Operation

**Figure 1:**
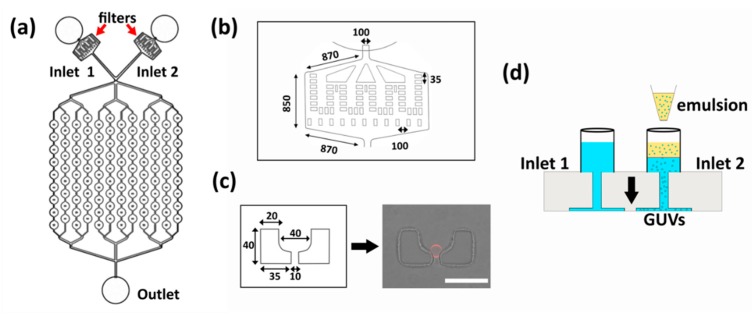
Device design and operation of the on-chip giant unilamellar vesicle (GUV) production. (**a**) Schematic representation of the device containing two inlets with filters, 108 traps arranged in 12 lines, and one outlet. (**b**) Schematic enlargement of the filter used in the design to prevent the entry of large particles or dirt. The minimum gap between the posts is 35 µm. Dimensions shown are in micrometers. (**c**) Schematic enlargement of a trap with dimensions in micrometers and a confocal image showing a single trapped GUV produced via the on-chip inverted emulsion method. DiIC_18_ fluorescence from the membranes is overlaid with the bright-field transmission image of the polydimethylsiloxane (PDMS) posts for visualization. (**d**) Schematic showing the side of the chip with two reservoirs where inlet 1 contains the glucose solution to balance the hydrostatic pressure from inlet 2, where the inverted emulsion method is performed. The arrow denotes the centrifugation step that is needed for the emulsion to pass through the interfacial lipid monolayer forming GUVs which accumulate at the bottom of the inlet 2 and later get trapped at the micro-posts.

The device design for on-chip production, trapping, and visualization of GUVs is shown in Figure 1a. The device includes two inlets (one for GUV production, the other for solution exchange), filters (Figure 1b) (to avoid unwanted blocking of the traps), 108 traps (in a 9 × 12 array), and a single outlet. An enlargement of the two-post trap design is shown in Figure 1c. One of the inlets is used to perform the inverted emulsion method and the other inlet is reserved for the addition of solutions during the subsequent experiments (Figure 1d). First, the chip is loaded with β-casein solution (2 mg/mL) via centrifugation (10 min at 900× *g*) and incubated for 30 min to coat the channel surfaces and avoid vesicle adhesion/rupture. This is followed by exchange with a glucose solution (80 µL) (centrifugation for 3 min at 900× *g*) and layering of the lipid–oil mixture (60 µL) in one of the reservoirs. After 30 min of incubation to form a lipid monolayer at the oil–water interface, a water-in-oil emulsion is produced by mixing 7 µL of isosmotic aqueous sucrose solution with 200 µL of the lipid–oil mixture in an Eppendorf tube. By mechanically agitating the suspension over an Eppendorf rack three times, water-in-oil (W/O) emulsion droplets are produced. Compared to microfluidic production of W/O droplets [35,36], the above method is time-effective and simple. While direct size tunability is not possible without the use of microfluidic droplet formation, here we demonstrate that with specific trap dimensions, partial size selection can be achieved (see below). Then, 50 µL of this emulsion is added to the reservoir containing the interface monolayer (Figure 1d). At this stage, equivalent volumes of glucose solution are added to the second reservoir to balance the hydrostatic pressure across the chip. As a last step, the chip is centrifuged at 180× *g* for 3 min to pass the emulsion through the monolayer and form GUVs. The resulting GUVs that are produced at the bottom of the inlet are drawn into the traps by applying a flow using a syringe pump operating in withdraw mode (Base 120, neMESYS pump, Cetoni, Korbussen, Germany). Once the single GUVs are trapped (Figure 1c; Video S1, Supplementary Materials), the reservoir hosting the inverted emulsion is blocked, and solution exchanges are performed through the other reservoir. The purpose of the second reservoir is to provide fluid exchange without the need to remove any oil from the first reservoir. Without this, residual oil can enter the traps, causing bursting and loss of GUVs (data not shown).

### 3.2. On-Chip Giant Unilamellar Vesicle (GUV) Production and Trapping Occupancy

**Figure 2:**
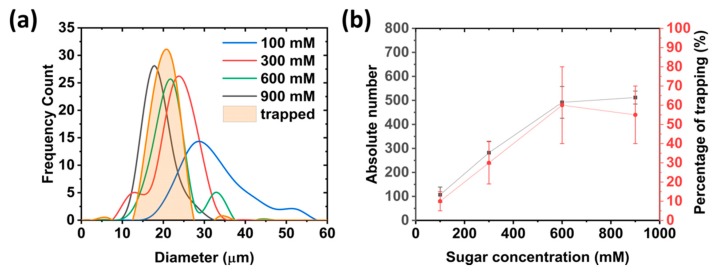
Optimization of the inverted emulsion method within the microfluidic device. (**a**) Distribution of GUV sizes produced in the chip with various sugar concentrations. Mean diameters were 31 ± 8, 25 ± 4, 21 ± 4, and 15 ± 3 µm for 100, 300, 600, and 900 mM respectively. For comparison, the size of the GUVs captured inside the microfluidic chip was 18 ± 4 µm in diameter. (**b**) Dependency of the yield of vesicles and the occupancy of the traps with respect to the sugar concentration. The percentage trapping occupancy was calculated by dividing the number of traps containing a GUV with that of total number of traps (108) multiplied by 100. Mean values are given with error bars taken from standard deviations (*n* = 3). Note that, for both cases, vesicles where counted manually.

The existence of a density difference between the solutions inside and outside of the GUVs is essential for their production using the inverted emulsion method [9,37]. Considering this, the on-chip production of giant vesicles was investigated using various concentrations of sugar solutions: 100 mM, 300 mM, 600 mM, and 900 mM. After centrifugation, we first analyzed the size of the produced GUVs at the inlet. We found that for lower sugar concentrations, the vesicles are larger in size but with more polydispersity (see Figure 2a). For the highest tested concentration of 900 mM, the average diameter was reduced to 15 ± 3 µm, giving some control over the size of the vesicles.

Next, we investigated the yield at the inlet, as well as the occupancy of the traps with the different sugar densities. Among the tested concentrations, both 600 mM and 900 mM conditions provided the highest yield (Figure 2b). There is a clear dependency of the vesicle yield on the sugar concentration (in agreement with previous studies [9]), which reaches a plateau at 600 mM. The GUVs were then drawn into the chip (5 µL/min for at least 15 min or until the inlet was free from vesicles) in order to examine the occupancy of the two-post traps (see Figure 1c for an example of a single trapped GUV). The occupancy followed a similar trend to that of the GUV yield, with more traps being filled with increasing sugar concentrations (Figure 2b). A maximum occupancy was reached at 60 ± 21% for 600 mM sugar. This was most likely due to the limited number of available GUVs formed at the inlet. A slight decrease at 900 mM, even though the yield was higher, was most likely due to a change in vesicle size from 21 ± 4 to 15 ± 3 µm. Trapping posts have been shown to capture a specific size range of GUVs [21], and here the mean diameter of the captured vesicles in the traps was 18 ± 4 µm (Figure 2a). Considering that the average gap between the two posts is 11 ± 0.9 µm, smaller vesicles (at 900 mM) are more likely to pass through trap and lower the overall probability of occupancy. In general, the trapping occupancy could, in principle, be improved by increasing the volume of emulsion and therefore the yield [9].

These data show that we are able to selectively tune the size of the GUVs under analysis. We note that this can also be achieved with microfluidic formation methods [38], however here we provide a much simpler set-up. Other sizes could be selected using different channel/trap dimensions [21]. For the proceeding experiments, a 600 mM sugar concentration was maintained for the production of GUVs to maximize the number of GUVs under observation within the traps.

### 3.3. Two-Layer Design for Multiple Experiments

**Figure 3:**
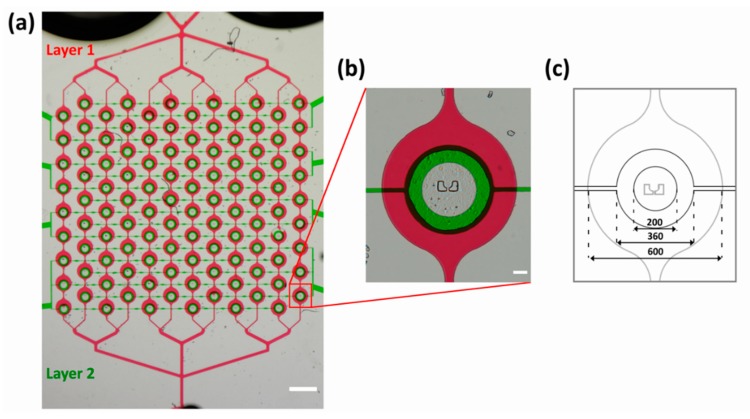
The two-layer microfluidic chip for multiple experiments. (**a**) Color bright-field microscopy image of the entire channel network. For visualization, the lower fluidic layer with traps contained magenta food dye, and the upper layer with valves contained green food dye. Scale bar corresponds to 1 mm. (**b**) Magnification of a single trap showing the alignment of the valve directly above the trapping posts. Scale bar corresponds to 100 µm. (**c**) Dimensions of the valve and the width of the underlying channel (dimensions are in micrometers).

To introduce more versatility, another layer was added to the two-inlet design described above. The second upper layer, situated above the previous fluidic layer, has circular valves over each of the 108 traps. Upon the application of 2 bar of pressure to the upper layer, the PDMS is deformed in a circular shape [21], making contact with the glass coverslip and therefore creating a sealed valve. Note that since PDMS is permeable to gas, direct usage of gas to pressurize the valves might result in the development of air bubbles within channels. To circumvent this problem, the top layer was filled with water via centrifugation at 900× *g* for 10 min. The purpose of the valves is to selectively provide or prevent fluidic access to the trapped vesicles. This can be used as an advantage to perform multiple independent experiments on a single batch of produced vesicles within the same chip. After the production and trapping of the GUVs, it is possible to close all the valves, thus isolating all of the GUVs from the external medium. By opening and closing specific valves, it is possible to selectively expose a sub-population of GUVs to a new external solution whilst still isolating the other GUVs from the new medium. The current design contains eight valves, thereby allowing eight independent experiments each with up to 12–18 traps or GUVs. The two-layer chip design can be seen in Figure 3a where the lower fluidic layer has the same design used for optimizing vesicle production (Figure 1). Figure 3b shows a higher magnification of a single trap with a closed valve. Note that the magenta food dye is excluded from the center of the trap, demonstrating the ability of the valves to isolate the trap and, therefore, the GUV from the rest of the channel network. Dimensions of the valve and chamber are provided in Figure 3c. A photograph of the final chip can be seen in Figure S1 in the Supplementary Materials.

**Figure 4:**
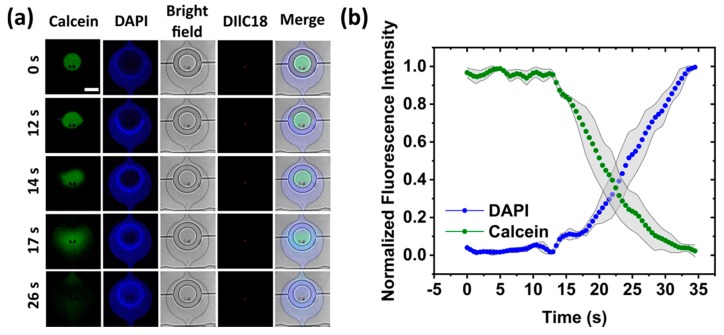
Solution exchange assay. (**a**) Confocal time-series of calcein and DAPI exchange after opening a set of valves. DiIC_18_ was used to stain the membrane of the GUVs, and bright-field images are included to visualize the valves. Scale bar 200 µm. (**b**) Mean fluorescence intensities of calcein and DAPI taken from the center of the valves (*n* = 3). Note that the valves were opened at 10 s. Shaded grey error bars are taken from the standard deviation.

A solution exchange experiment was performed on the trapped GUVs to demonstrate the ability of the two-layer design to deliver new solutions to a specific population of trapped GUVs (see Video S2, Supplementary Materials). Calcein dye at a concentration of 20 µM in 600 mM glucose solution was added to the second reservoir and flushed into the chips at a flow rate of 2 µL/min with the valves opened. Once the entire device was filled with calcein, the valves were closed (2 bar) and the solution in the chip was replaced with DAPI (20 µM in 600 mM glucose solution) using the same procedure. A single set of valves were then opened (back to 0 bar) with no flow, and a time series was recorded of the solution exchange process (Figure 4a). As can be seen from Figure 4b, complete fluidic exchange occurred within 20 s as DAPI replaced calcein. Here, the solution exchange was purely diffusive as no flow was present, but faster times can be achieved with flow (see Video S3, Supplementary Materials). This assay was performed on a single set of valves demonstrating the ability of the device to produce, trap, and perform solution exchange on a sub-population of GUVs only. Next, we present a series of tests to demonstrate that multiple experiments can be conducted.

#### 3.3.1. Membrane Transport Assay

**Figure 5:**
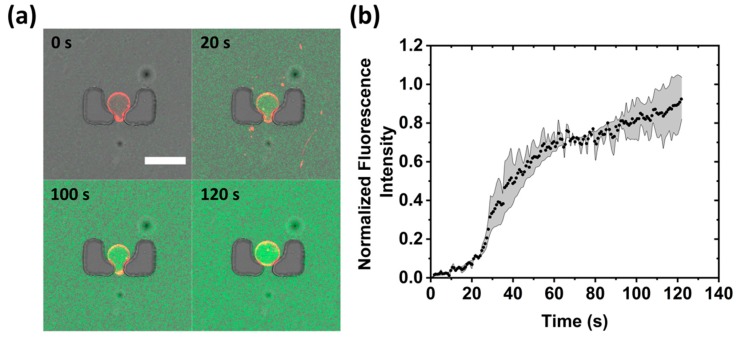
Calcein dye transport assay using alpha-hemolysin membrane pore protein. (**a**) Confocal time-series of a trapped GUV (red) after exposure to the protein and calcein solution (green). Scale bar 50 µm. (**b**) Mean fluorescence intensity of calcein inside the GUVs over time (*n* = 3). Shaded grey error bars are taken from the standard deviation.

A calcein transport assay is presented using alpha-hemolysin membrane pore protein to demonstrate the ability to perform multiple experiments on the same device. A solution of 2.5 µg/mL alpha-hemolysin together with 20 µM calcein in 600 mM glucose was flushed through the device for a period of 10 min at a flow rate of 2 µL/min while the valves of two rows were opened and the rest of the valves were closed. From the data presented in Figure 5a, it is evident that the GUV was porous within a few seconds as calcein can be seen within the lumen. Figure 5b shows the profile of calcein inside the GUV over time (see Video S4, Supplementary Materials). Successful formation of a membrane pore also confirms the unilamellarity of the membranes. This result shows that by using a two-inlet design, one can perform entire GUV production, trapping, and analysis within the same chip without requiring the removal of the residual oil left over in the production step. Importantly, this experiment was performed independently from the solution exchange above, but on the same device.

#### 3.3.2. Membrane Permeabilization and Rupture

**Figure 6:**
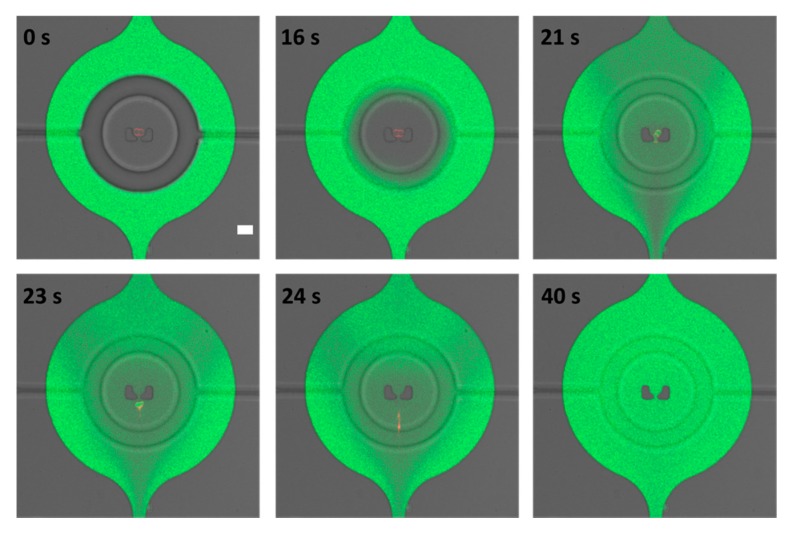
Membrane permeabilization and rupture. A confocal time-series of GUV (red) permeation and rupture using 0.1% Triton X-100 solution together with 20 µM calcein (green). Note that the valve was opened at 16 s. Scale bar 50 µm.

GUVs that were isolated from the alpha-hemolysin assay were then used for a membrane permeabilization and rupture test. After exchanging the external solution with 0.1% Triton X-100 and 20 µM calcein in 600 mM glucose, these isolated GUVs were exposed by opening the valves. Note that even after more than an hour of valve pressure and the constant presence of multiple solutions, the GUVs were perfectly stable and isolated. After opening of the valves, and in the presence of 2 µL/min flow, the exchange of fluid happened within 10 s (see Video S3, Supplementary Materials). Figure 6 shows the permeabilization of the lipid bilayer by Triton X-100 via calcein dye penetration into the lumen of a GUV at 21 s (see Video S5, Supplementary Materials) which is followed by rupture in the next 2 s. It is well known that Triton X-100 can solubilize lipids [39], and these data show that before complete solubilization and rupture, pores are formed which are large enough for small dye molecules to cross the membrane. This last assay shows that not only can formation and trapping be conducted on a single platform, but multiple separate experiments are also feasible.

## 4. Conclusions

Even though multipurpose integrated devices for GUVs studies are much needed for fields such as synthetic biology, instances of them in the literature are rare. The microfluidic method presented in this work is able to address this shortcoming due to its ability to produce, trap, and perform multiple experiments in one single platform. GUVs are first formed via an on-chip inverted emulsion method with optimized sugar solutions set at 600 mM and are then introduced into an array of 108 traps designed to capture single GUVs. The W/O emulsion is formed off-line in bulk so the resulting GUVs formed at the device inlet are polydisperse in size. However, owing to the specific height (20 µm) and gap between the two trapping posts (11 µm), this results in the size-selective capture of GUVs with an average diameter of 18 ± 4 µm. Furthermore, due to the presence of integrated valves, specific sub-sets of these trapped vesicles can then be isolated from the rest of the channel network and experimented on separately. We demonstrated this by performing (i) a solution exchange test, (ii) a membrane transport assay, and (iii) a permeation/rupture experiment all on the same device. Importantly, all of these experiments were all conducted on the same batch of vesicles produced in the same device, thus eliminating the possibility of batch-to-batch differences between experiments. Another advantage of forming GUVs and delivering new solutions on a microfluidic chip is that the required sample volumes are greatly reduced compared to bulk methods. Being able to fully exchange external GUVs solutions is not possible using bulk approaches. Here, we show that this is not only possible with our platform, but it can also be performed in less than 10 s. We foresee the ability of this integrated device to perform multiple experiments on a single batch of on-chip produced vesicles being applicable to membrane biophysical studies, as well as the bottom-up construction of artificial cells.

## Supplementary Materials

The following are available online at https://www.mdpi.com/2072-666X/11/3/285/s1, Figure S1: Photographs of the fully assembled two layer device. Video S1: capture of a GUV into the two post trap. Video S2: diffusive fluid exchange after opening the valve. Video S3: fast fluid exchange. Video S4: alpha-hemolysin induced membrane transport. Video S5: membrane permeation and rupture assay.

## References

[1] Schwille P., Spatz J.P., Landfester K., Bodenschatz E., Herminghaus S., Sourjik V., Erb T.J., Bastiaens P.I.H., Lipowsky R., Hyman A.A. (2018). MaxSynBio: Avenues Towards Creating Cells from the Bottom Up. Angew. Chem. Int. Ed..

[2] Robinson T. (2019). Microfluidic Handling and Analysis of Giant Vesicles for Use as Artificial Cells: A Review. Adv. Biosyst..

[3] Weinberger A., Tsai F.-C., Koenderink G.H., Schmidt T., Itri R., Meier W., Schmatko T., Schröder A., Marques C. (2013). Gel-Assisted Formation of Giant Unilamellar Vesicles. Biophys. J..

[4] Yandrapalli N., Lubart Q., Tanwar H.S., Picart C., Mak J., Muriaux D., Favard C. (2016). Self assembly of HIV-1 Gag protein on lipid membranes generates PI(4,5)P2/Cholesterol nanoclusters. Sci. Rep..

[5] Montes L.-R., Alonso A., Goñi F.M., Bagatolli L.A. (2007). Giant unilamellar vesicles electroformed from native membranes and organic lipid mixtures under physiological conditions. Biophys. J..

[6] Carvalho K., Ramos L., Roy C., Picart C. (2008). Giant Unilamellar Vesicles Containing Phosphatidylinositol(4,5)bisphosphate: Characterization and Functionality. Biophys. J..

[7] Dimova R., Marques C. (2019). The Giant Vesicle Book.

[8] Trantidou T., Friddin M.S., Elani Y., Brooks N., Law R., Seddon J.M., Ces O. (2017). Engineering Compartmentalized Biomimetic Micro- and Nanocontainers. ACS Nano.

[9] Moga A., Yandrapalli N., Dimova R., Robinson T. (2019). Optimization of the Inverted Emulsion Method for High-Yield Production of Biomimetic Giant Unilamellar Vesicles. ChemBioChem.

[10] Pautot S., Frisken B.J., Weitz D.A. (2003). Production of Unilamellar Vesicles Using an Inverted Emulsion. Langmuir.

[11] Love C., Steinkühler J., Gonzales D.T., Yandrapalli N., Robinson T., Dimova R., Tang T.-Y.D. (2020). Reversible pH responsive coacervate formation in lipid vesicles activates dormant enzymatic reactions. Angew. Chem..

[12] Weiss M., Frohnmayer J.P., Benk L.T., Haller B., Janiesch J.-W., Heitkamp T., Börsch M., Lira R.B., Dimova R., Lipowsky R. (2017). Sequential bottom-up assembly of mechanically stabilized synthetic cells by microfluidics. Nat. Mater..

[13] Ugrinic M., Demello A.J., Tang T.-Y.D. (2019). Microfluidic Tools for Bottom-Up Synthetic Cellularity. Chem.

[14] Elani Y. (2016). Construction of membrane-bound artificial cells using microfluidics: a new frontier in bottom-up synthetic biology. Biochem. Soc. Trans..

[15] Sato Y., Takinoue M. (2019). Creation of Artificial Cell-Like Structures Promoted by Microfluidics Technologies. Micromachines.

[16] Stano P., Carrara P., Kuruma Y., De Souza T.P., Luisi P.L. (2011). Compartmentalized reactions as a case of soft-matter biotechnology: synthesis of proteins and nucleic acids inside lipid vesicles. J. Mater. Chem..

[17] Hadorn M., Boenzli E., Hotz P.E., Hanczyc M. (2012). Hierarchical Unilamellar Vesicles of Controlled Compositional Heterogeneity. PLoS ONE.

[18] Natsume Y., Wen H.-I., Zhu T., Itoh K., Sheng L., Kurihara K. (2017). Preparation of Giant Vesicles Encapsulating Microspheres by Centrifugation of a Water-in-oil Emulsion. J. Vis. Exp..

[19] Kazayama Y., Teshima T., Osaki T., Takeuchi S., Toyota T. (2015). Integrated Microfluidic System for Size-Based Selection and Trapping of Giant Vesicles. Anal. Chem..

[20] Estes D.J., Lopez S.R., Fuller A.O., Mayer M. (2006). Triggering and Visualizing the Aggregation and Fusion of Lipid Membranes in Microfluidic Chambers. Biophys. J..

[21] Robinson T., Kuhn P., Eyer K., Dittrich P.S. (2013). Microfluidic trapping of giant unilamellar vesicles to study transport through a membrane pore. Biomicrofluidics.

[22] Yamada A., Lee S., Bassereau P., Baroud C.N. (2014). Trapping and release of giant unilamellar vesicles in microfluidic wells. Soft Matter.

[23] Yandrapalli N., Robinson T. (2019). Ultra-high capacity microfluidic trapping of giant vesicles for high-throughput membrane studies. Lab Chip.

[24] Kuribayashi K., Tresset G., Coquet P., Fujita H., Takeuchi S. (2006). Electroformation of giant liposomes in microfluidic channels. Meas. Sci. Technol..

[25] Paterson D.J., Reboud J., Wilson R., Tassieri M., Cooper J.M. (2014). Integrating microfluidic generation, handling and analysis of biomimetic giant unilamellar vesicles. Lab Chip.

[26] Wang Z., Hu N., Yeh L.-H., Zheng X., Yang J., Joo S.W., Qian S. (2013). Electroformation and electrofusion of giant vesicles in a microfluidic device. Colloids Surf. B.

[27] Matosevic S., Paegel B. (2011). Stepwise Synthesis of Giant Unilamellar Vesicles on a Microfluidic Assembly Line. J. Am. Chem. Soc..

[28] Schaich M., Cama J., Al Nahas K., Sobota D., Sleath H., Jahnke K., Deshpande S., Dekker C., Keyser U.F. (2019). An Integrated Microfluidic Platform for Quantifying Drug Permeation across Biomimetic Vesicle Membranes. Mol. Pharm..

[29] Al Nahas K., Cama J., Schaich M., Hammond K., Deshpande S., Dekker C., Ryadnov M.G., Keyser U.F. (2019). A microfluidic platform for the characterisation of membrane active antimicrobials. Lab Chip.

[30] Petit J., Polenz I., Baret J.-C., Herminghaus S., Bäumchen O. (2016). Vesicles-on-a-chip: A universal microfluidic platform for the assembly of liposomes and polymersomes. Eur. Phys. J. E.

[31] Deshpande S., Caspi Y., Meijering A.E.C., Dekker C. (2016). Octanol-assisted liposome assembly on chip. Nat. Commun..

[32] Shum H.C., Lee D.-J., Yoon I., Kodger T., Weitz D.A. (2008). Double Emulsion Templated Monodisperse Phospholipid Vesicles. Langmuir.

[33] Deng N.-N., Yelleswarapu M., Zheng L., Huck W.T. (2016). Microfluidic Assembly of Monodisperse Vesosomes as Artificial Cell Models. J. Am. Chem. Soc..

[34] Kubsch B., Robinson T., Steinkühler J., Dimova R. (2017). Phase Behavior of Charged Vesicles Under Symmetric and Asymmetric Solution Conditions Monitored with Fluorescence Microscopy. J. Vis. Exp..

[35] Nishimura K., Suzuki H., Toyota T., Yomo T. (2012). Size control of giant unilamellar vesicles prepared from inverted emulsion droplets. J. Colloid Interface Sci..

[36] Hu P.C., Li S., Malmstadt N. (2011). Microfluidic fabrication of asymmetric giant lipid vesicles. ACS Appl. Mater. Interfaces.

[37] Hamada T., Miura Y., Komatsu Y., Kishimoto Y., Vestergaard M., Takagi M. (2008). Construction of Asymmetric Cell-Sized Lipid Vesicles from Lipid-Coated Water-in-Oil Microdroplets. J. Phys. Chem. B.

[38] Teh S.-Y., Khnouf R., Fan Z.H., Lee A.P. (2011). Stable, biocompatible lipid vesicle generation by solvent extraction-based droplet microfluidics. Biomicrofluidics.

[39] Mattei B., Lira R.B., Perez K.R., Riske K.A. (2017). Membrane permeabilization induced by Triton X-100: The role of membrane phase state and edge tension. Chem. Phys. Lipids.

