# Supplementary material for: On-Chip Inverted Emulsion Method for Fast Giant Vesicle Production, Handling, and Analysis
Source: Micromachines (Basel). 2020 Mar 10;11(3):285. doi: 10.3390/mi11030285 (PMC7142477; doi:10.3390/mi11030285)
Supplement: Supplementary file 1 [file micromachines-11-00285-s001.zip › micromachines-725847-supplementary materials-for proof/micromachines-725847-supplementary-for proof.docx]

Supplementary Materials

On-Chip Inverted Emulsion Method for Fast Giant Vesicle Production, Handling, and Analysis

Naresh Yandrapalli, Tina Seemann and Tom Robinson


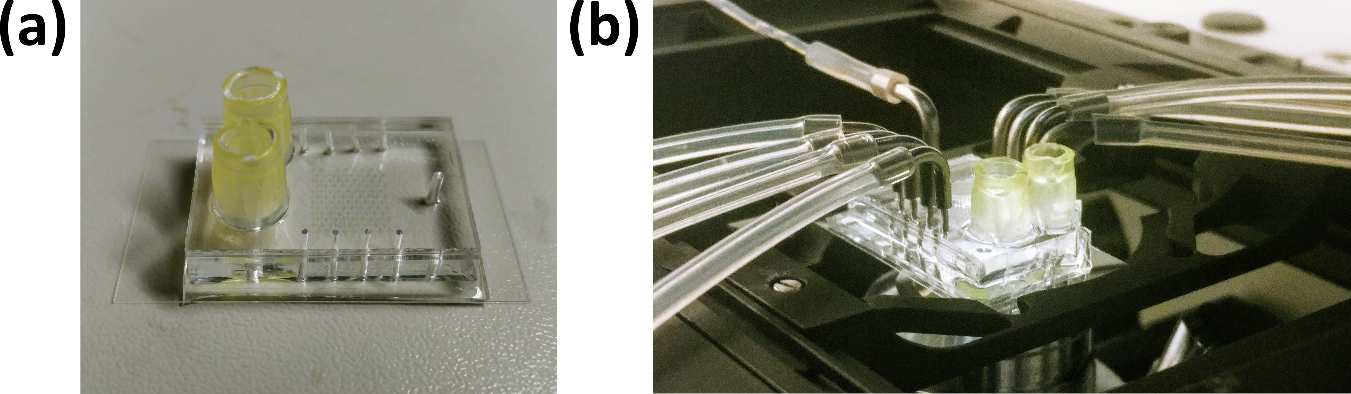


**Figure S1.** Photographs of the fully assembled two layer device. (**a**) Showing the 2 reservoirs (**left**), 8 pressure inlets for the valves (**middle**), and 1 outlet for fluidic control (**right**). (**b**) Photograph of the same device on the confocal microscope during the experiment with pressure inlets and outlets connected to external devices.

Video S1: Capture of a GUV into the Two Posts Trap.

Time series demonstrating that GUVs produced on-chip can be captured in the same microfluidic device with two post traps. Scale bar corresponds to 50 µm.

Video S2: Diffusive Fluid Exchange after Opening the Valve.

Time series showing that in the absence of flow, the exchange of the fluids occurs within less than a minute. This is useful for experiments where the flow is detrimental or not necessary for observation. Scale bar corresponds to 50 µm.

Video S3: Fast Fluid Exchange.

Time series showing that in the presence of 2µL/min flow, solution exchange can be performed within less than 10 seconds. Scale bar corresponds to 50 µm.

Video S4: Alpha-Hemolysin Induced Membrane Transport.

Time series of alpha-hemolysin membrane pore protein addition and the subsequent transport of the calcein dye into the lumen of the GUV. Scale bar corresponds to 20 µm.

Video S5: Membrane Permeation and Rupture Assay.

Showing the membrane permeabilization and final GUV rupture by 0.1% Triton X-100. Scale bar corresponds to 50 µm.

| 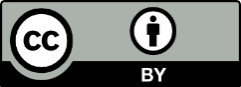 | © 2020 by the authors. Submitted for possible open access publication under the terms and conditions of the Creative Commons Attribution (CC BY) license (http://creativecommons.org/licenses/by/4.0/). |
| --- | --- |
